# The Distribution, Expression Patterns and Functional Analysis of NR1D1 and NR4A2 in the Reproductive Axis Tissues of the Male Tianzhu White Yak

**DOI:** 10.3390/ani11113117

**Published:** 2021-10-31

**Authors:** Lijun Dai, Quanwei Zhang, Jun Shi, Xu Bai, Xiaoxiao An, Bohao Zhang, Yong Zhang, Xingxu Zhao

**Affiliations:** 1 College of Life Science and Biotechnology, Gansu Agricultural University, Lanzhou 730070, China; d1312496231@163.com (L.D.); sj18034650439@163.com (J.S.); b1820313351@126.com (X.B.); a1732036832@163.com (X.A.); zhangy@gsau.edu.cn (Y.Z.); 2 Gansu Key Laboratory of Animal Generational Physiology and Reproductive Regulation, Lanzhou 730070, China; zhangbhgs@163.com; 3 College of Veterinary Medicine, Gansu Agricultural University, Lanzhou 730070, China

**Keywords:** NR1D1, NR4A2, yak, reproduction axis, sterol hormone

## Abstract

**Simple Summary:**

The reproductive function and behaviors of male animals are regulated by HPG tissues that are responsible for hormone synthesis and secretion. The NRs, as a kind of transcription factor, are recruited to some ligands, to convey and translate signals for regulation of hormone-related gene expression. Understanding the distribution, expression and function of NRs in reproductive axis tissues is vital for elucidating the molecular mechanisms of animal reproduction. NR1D1 and NR4A2, members of the NR superfamily, are important receptors of hormones, but lacking in identified target genes and related molecular mechanisms, particularly in yaks. In the present study, we found that NR1D1 and NR4A2 proteins were present in all yak HPG tissues, particularly in the adenohypophysis, Leydig cells, principal cells and cilia of epididymis. Yak testicular tissues from animals of different ages confirmed that the expression levels of NR1D1 and NR4A2 were up-regulated or down-regulated with the maturation or decline of sexual function. These results suggested that NR1D1 and NR4A2, as important mediators, are involved in the regulation of steroid hormones metabolism in male yaks.

**Abstract:**

Nuclear hormone receptors NR1D1 and NR4A2 play important roles in the synthesis and metabolism of hormones that are thought to be strictly regulated by the hypothalamus-pituitary-gonad axis (HPG) tissues via gene expression. However, in the yak, the function and regulatory mechanisms of NR1D1 and NR4A2 are not clearly understood. The current study is aimed to investigate the expression patterns, distribution and functions of these two receptors in HPG tissues in male Tianzhu white yaks. Immunohistochemical staining showed NR1D1 and NR4A2 proteins were present in all yak HPG tissues with differential expression patterns and degrees of staining, particularly in Leydig cells that were strongly positive in accordance with the immunofluorescence results. qRT-PCR and Western blot results suggested that the highest expression levels of *NR1D1* and *NR4A2* mRNA were present in the hypothalamus, while the expression levels of NR1D1 and NR4A2 proteins were higher in the testis and epididymis than in the hypothalamus or pituitary gland. In addition, expression levels of *NR1D1* and *NR4A2* mRNA and protein in testicular tissues differed by age. Expression levels were significantly higher at 6 years of age. Gene ontology (GO) and pathway analysis enrichment revealed that NR1D1 may directly regulate the synthesis and metabolism of steroid hormones via interaction with different targets, while NR4A2 may indirectly regulate the synthesis and metabolism of steroid hormones. These results showed that NR1D1 and NR4A2, as important mediators, are involved in the regulation of male yak reproduction, and especially of steroid hormones and androgen metabolism. These results will be helpful for the further understanding of the regulatory mechanisms of NR1D1 and NR4A2 in yak reproduction.

## 1. Introduction

The Tianzhu white yak is a rare and unique domesticated animal from the Tianzhu Tibetan Autonomous County (Wuwei City, Ganzu Province, China), famous for its full-bodied white color [1,2]. Like cattle and other yak breeds, it plays important roles in the normal life of people living on the plateau, such as providing meat, milk and wool, and their ability to pack goods and materials and for riding [3]. The population of the Tianzhu white yak is no more than 100,000, only forming 0.63% of the global yak population (16 million) [4]. Their low population is due mainly to their poor productive and reproductive performance. In some yak production areas of China, the relatively short duration of oestrus (12–36 h) and oestrus cycle (19–21 d) of the female yak and irrational mating or inbreeding within the male yaks result in only 50% conception rates [5]. Thus, there is a pressing need to clarify the yak reproductive mechanisms to improve reproductive performance. Reproductive hormones strictly regulate the reproductive process in animals [6,7], and the complex functions and regulatory roles of hormones have been in-depth for many years [8,9]. However, there is a lack of consensus regarding the changes and regulatory mechanisms of reproductive hormones as some mechanisms remain.

Nuclear hormone receptors (NRs) play important roles in many biological processes, including metabolism, reproduction and morphogenesis [10]. A classic example of which is the regulation of reproductive hormones. The NR superfamily, which includes steroid hormone, thyroid hormone, retinoic acid, vitamin D, oxysterol and fatty acid derivative classes of receptors [10,11], are generally recruited to ligand-bound nuclear receptors and are responsible for gene expression and regulation. It is well known that steroid hormones contain sex hormones such as testosterone, progesterone and estradiol [12], which are also strictly mediated by a series of enzymes or genes. Nuclear receptors play an essential role in the regulation of reproduction, development and growth by regulating enzymes and genes, including the testis receptor, androgen receptor and thyroid hormone receptor. However, the enzymes or genes regulating the synthesis of steroid hormones are not entirely known. Nuclear receptor subfamily 1, group D, member 1 (NR1D1) and nuclear receptor subfamily 4, group A, member 2 (NR4A2), two of the transcription factors belonging to the nuclear receptor superfamily, are important receptors of hormones [13,14]. NR1D1, also known as REV-ERB-α, an auxiliary component of the circadian clock system [14], is responsible for some biorhythm regulation. Reproductive hormone secretion rhythm, one of these typical examples, is accurately controlled by the reproductive axis, the hypothalamus-pituitary-gonad axis (HPG). Whether or not NR1D1 is expressed in HPG tissues could be direct evidence proving its function or role in reproductive hormone synthesis. It has been demonstrated that NR4A2 can recruit and activate transcription of the genes Steroidogenic Acute Regulatory protein (StAR) or 3β-hydroxysteroid dehydrogenase (3β-HSD) in Leydig cells [15]. Leydig cells produce some sex hormones, including testosterone and dihydrotestosterone, which are crucial for the male fetus, sexual behavior, sex accessory gland development and function, and initiation and maintenance of spermatogenesis [16,17]. 

The evidence showed that NR1D1 and NR4A2 could be important regulatory factors or recruit hormones for reproduction and reproductive hormones. However, the relationship or interaction network between NR1D1, NR4A2 and the receptors regulating androgen synthesis in yak testes remains unclear. Therefore, the goals of the present study were to perform a preliminary exploration of the expression patterns, expression position and potential functions of NR1D1 and NR4A2 in steroid hormone and androgen synthesis and metabolism and to provide the basis of knowledge for the further study of their mechanisms.

## 2. Materials and Methods

### 2.1. Sample Preparation and Collection

Fresh HPG tissues, including the hypothalamus, hypophysis, epididymis (caput, corpus and cauda) and testis tissues, from adult male yaks (4 years old, n = 6) were obtained immediately after slaughter in Tianzhu county (Wuwei City, Gansu Province, China). Testicular tissues were also collected from yaks of different ages (2, 4, 6 and 8 years old, n = 6). All samples used in the present study were collected during the yak breeding season (August to September). Parts of the tissues were fixed by 4% paraformaldehyde for morphological observation and subcellular location analysis using Hematoxylin–Eosin (H&E), immunohistochemistry (IHC) and immunofluorescence (IF) staining. Parts of the tissues were stored immediately at −80 °C for mRNA and protein expression pattern analysis using quantitative real-time polymerase chain reaction (qRT-PCR) and Western blot. All samples were collected in strict accordance with the ethical guidelines approved by the Animal Care Commission of Gansu Agricultural University (code GSAU-Eth-LST-2021-003).

### 2.2. H&E staining

Morphologic observation of the fixed HPG tissues was performed using H&E staining. The fixed HPG tissues were applied to morphologic observation using H&E staining. The fixed HPG tissues were embedded into paraffin (Solarbio, Beijing, China) and cut into 5 μm thickness sections using a microtome (Lecia, Weztlar, Germany). The sections were deparaffinized in xylene and rehydrated in an ethanol gradient. H&E staining was carried out as described previously [18]. The images were captured using an Olympus BX53M microscope (Olympus, Tokyo, Japan).

### 2.3. IHC Staining and IF Assay

NR1D1 and NR4A2 proteins were detected using an immunohistochemical standard avidin–biotin–peroxidase method of the ABC staining system (Bioss, Beijing, China). Antigen retrieval was performed by heating in a microwave oven (750 W for 10 min) and cooling slowly to room temperature. Endogenous catalase deactivation was performed by immersion of the slides in 0.3% (*v*/*v*) hydrogen peroxide for 30 min at room temperature. After washing with PBS, reagent A was added and incubated at room temperature for 30 min. Rabbit polyclonal anti-NR1D1 and anti-NR4A2 (1:300, Bioss) was used to capture proteins and phosphate buffer solution (PBS, Solarbio, Beijing, China) was used as a negative control, incubated at 4 °C overnight. The slides where incubated with the secondary antibody (reagent B) at 37° for 30 min. The slides where then incubated with reagent C (37° for 30 min), followed by DAB color development and hematoxylin redyeing. IHC staining was carried out as described previously [1,2,18]. IF staining with NR1D1, NR4A2 and 3β-HSD antibodies was performed for co-localization analysis of Leydig cells in epididymis (caput, corpus and cauda) and testis tissues as previously described [19,20]. 3β-HSD protein (rabbit polyclonal anti-3β-HSD, Bioss) was used as a marker of testicular Leydig cells [21]. Immunofluorescent staining was performed similarly to IHC, except that the secondary antibody differed. Most steps of the IF followed those of IHC staining except for the secondary antibody. After incubation with the primary antibody, samples were incubated with the appropriate HRP-conjugated secondary antibody (CY3 for NR1D1, FITC for NR4A2 and CY5 for 3β-HSD, Bioss) at a 1:250 dilution. Nuclei were counterstained with a 10 μg/mL DAPI. Images were captured using an Olympus fluorescence microscope (Olympus, Tokyo, Japan). All immuno-staining assays were performed at least in triplicate.

### 2.4. RNA Isolation and cDNA Synthesis

Total RNA was extracted from the yak HPG tissues and testis tissues using a FastPure RNA isolation kit (Vazyme, Nanjing, China), following the manufacturer’s instructions, and used for cDNA synthesis. The RNA concentration was quantified on a NanoDrop-8000 (Thermo, Waltham, MA, USA) and RNA integrity was assessed by denaturing formaldehyde agarose gel (1 %) electrophoresis (Biowest Regular Agarose, Castropol, Spain). 1 μg of total RNA was subjected to reverse transcription to single-stranded cDNA using a BioTeke Thermo RT Kit (Bioteke, Beijing, China). The reverse transcription PCR reaction was 48 °C for 50 min, followed by 70 °C for 10 min. The cDNA synthesis was performed in a 20 μL reaction volume containing 1 μg total RNA, 1 μL Oligo dT or Random Primer (50 μM), dNTP Mixture (10 μM), Thermo M-MLV (200 U/μL), RNase Inhibitor (40 U/μL), 4μL 5× first-strand buffer and an appropriate volume of ultrapure Millipore water (Invitrogen, Carlsbad, CA, USA).

### 2.5. qPCR

Relative expression levels of *NR1D1* and *NR4A2* in yak HPG and testis tissues were measured using qPCR. qPCR primers were designed using the Premier 5.0 software [1] and were synthesized by Qinke Biotech Co. Ltd. (Shanxi, China). Primer sequences are shown in Table S1. qPCR was performed on a LightCycler 96 real-time system (Roche, Switzerland) using a 2 μL cDNA template and SYBR premix Ex Taq™ II in a 20 μL reaction volume according to the manufacturer’s instructions. *β-actin* was used as an endogenous control. A denaturation step was run for one cycle at 95 °C for 30 s. The annealing step was run for 45 cycles at 95 °C for 5 s and 59 °C for 30 s. All PCR reactions were performed in triplicate. The expression of *NR1D1* and *NR4A2* mRNA in the hypothalamus (4 years old) or testis tissue (2 years old) were used as controls. The results were calculated using the 2^−ΔΔCT^ method [1,2].

### 2.6. Western Blot

The relative expression patterns of NR1D1 and NR4A2 proteins in HPG from the adult yaks and testis tissues from animals of different ages were examined using Western blot. Total protein was extracted from 100 mg of each tissue sample using RAPI (Solarbio, Beijing, China). Protein concentration was determined using a BCA kit (Solarbio). 100 μg of total protein samples were electrophoresed in a sodium dodecyl sulfate polyacrylamide gel (SDS-PAGE) for Western blot analysis. The blots were electro-transferred onto a PVDF membrane (Millipore CAT, Billerica, MA, USA), and blocked with Tris-HCl buffer (Solarbio) containing 5 % (*w*/*v*) non-fat milk (Solarbio, Beijing, China) for 2 h at room temperature. The membranes were incubated at 4 °C overnight with rabbit monoclonal anti-NR1D1 (1:300), anti-NR4A2 (1:300), and anti-β-actin (1:4000, Bioss, Beijing, China) primary antibodies. The subsequent procedures were carried out as described previously [22]. All immunoblot assays were performed at least in triplicate. Optical densities of the bands were quantified and scanned using Image-Pro Plus 6.0 (Media Cybernetics Co., Rockville, USA). The expression level of β-Actin was used as an endogenous control. The expression patterns of NR1D1 and NR4A2 proteins in the hypothalamus (4 years old) or testis tissue (2 years old) were used as controls. Data were presented as mean ± SD.

### 2.7. Protein and Protein Interaction Network

In order to better understand the functional and regulatory roles of NR1D1 and NR4A2 in male yak reproductive hormone biosynthesis, the protein-protein interaction (PPI) networks were constructed using the STING v 10.0 the candidate proteins involved in sterol hormone biosynthesis [12], such as androgen receptor (AR), StAR and cytochrome P450, family 17 subfamily A, polypeptide 1 (CYP17A1) (detailed in Table S2) using STRING v 10.0 database (online, https://www.string-db.org/ accessed on 30 October 2021) [23] and Cytoscape 2.8.1 software [24]. Further Gene Ontology (GO) and Kyoto Encyclopedia of Genes and Genome (KEGG) network analyses were performed using Clue-go and Ingenuity pathway analysis (IPA) (Ingenuity Systems, www.ingenuity.com, accessed on 21 July 2021).

### 2.8. Statistical Analysis

The data were presented as the mean ± SD, unless otherwise indicated. Statistical analysis was performed using SPSS version 21.0 (SPSS Inc., Chicago, IL, USA). The qPCR and Western blot data were analyzed using the Student’s test (between two groups) or one-way ANOVA analysis (within multiple groups). The graphs were drawn using Prism 5.0 (GraphPad Software Inc., San Diego, CA, USA). *p* < 0.05 was considered as statistically significantly different.

## 3. Results

### 3.1. Morphologic Observation of Adult Yak HPG Tissues

**Figure 1:**
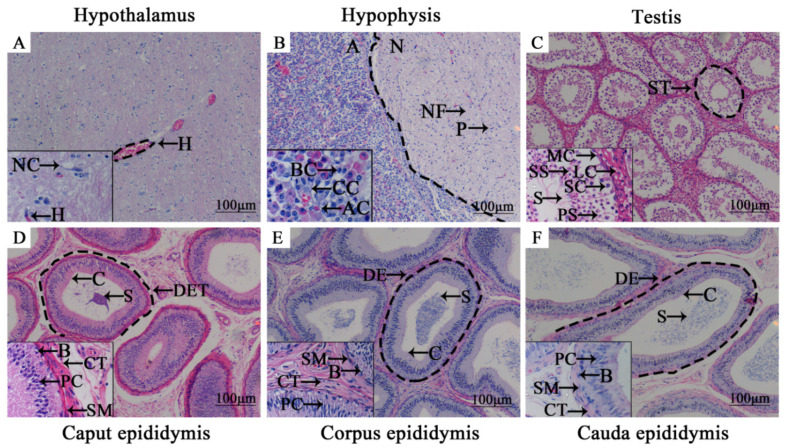
H&E staining of HPG tissues of an adult male yak (4 years old). （**A**–**F**） H&E staining of hypothalamus, hypophysis, testis, caput epididymis, corpus epididymis and cauda epididymis tissues, respectively (100×). The box in the lower left corner is a partial enlarged view (400×)**.** H, hematocyte; NC, neurogliocyte; A, adenohypophysis; N, neurohypophysis; P, pituicyte; AC, acidophilic cells; BC, basophilic cells; CC, chromophobe cells; NF, nerve fibers; ST, seminiferous tubule; MC, myoid cells; LC, interstitial cells; SC, spermatogonium; PS, primary spermatocyte; SS, secondary spermatocyte; S, spermatozoon; DET, ductuli efferentes testis; B, basal cells; PC, principal cells; SM, smooth muscle cells; CT, connective tissue; DE, ductus epididymidis.

Histomorphology of the yak HPG tissues were observed using H&E staining and the results showed that structural organization and cells could be observed clearly (Figure 1). Glial cells, an important part of the nervous system, could be observed in yak hypothalamus with various shapes. The adenohypophysis, composed of acidophilic cells, basophilic cells, chromophobe cells and neurohypophysis with the typical nerve cells including nerve fibers and pituicyte, were also obviously present in the yak hypophysis, which was separated by a clear dividing line. In yak testicular tissues, spermatogenic tubules and mesenchymal cells were observed. The seminiferous tubules were arranged densely, and there were flat, contractile myoid cells on the outside of the basement membrane of each tubule. The connective tissue between adjacent tubules contained polygonal Leydig cells. From the basement membrane, the spermatogenic cells and spermatogonia at various developmental stages could be seen. More sperm cells, late metamorphosis sperm cells and sperm were seen on the abluminal surface. The epithelium of the epididymis wall of the yak is composed mainly of principal cells, basal cells and smooth muscle cells. There were three to four layers of structure that could be seen in each part of the epithelium. A large number of mature sperm were distributed on the lumen surface, with the main cells are distributed in a tall column and the smooth muscle cell nuclei were long spindle-shaped and surrounded the outer periphery of each epididymal tube propria. Basal cells were in a single layer, distributed at the bottom of the propria of each epididymal duct. The cilia in the lumen of the caput and the corpus epididymis were longer, while the cilia in the lumen of the cauda epididymis were shorter, with a clear structure.

### 3.2. Subcellular Location Analysis of NR1D1 and NR4A2 in Adult Yak HPG Tissues

**Figure 2:**
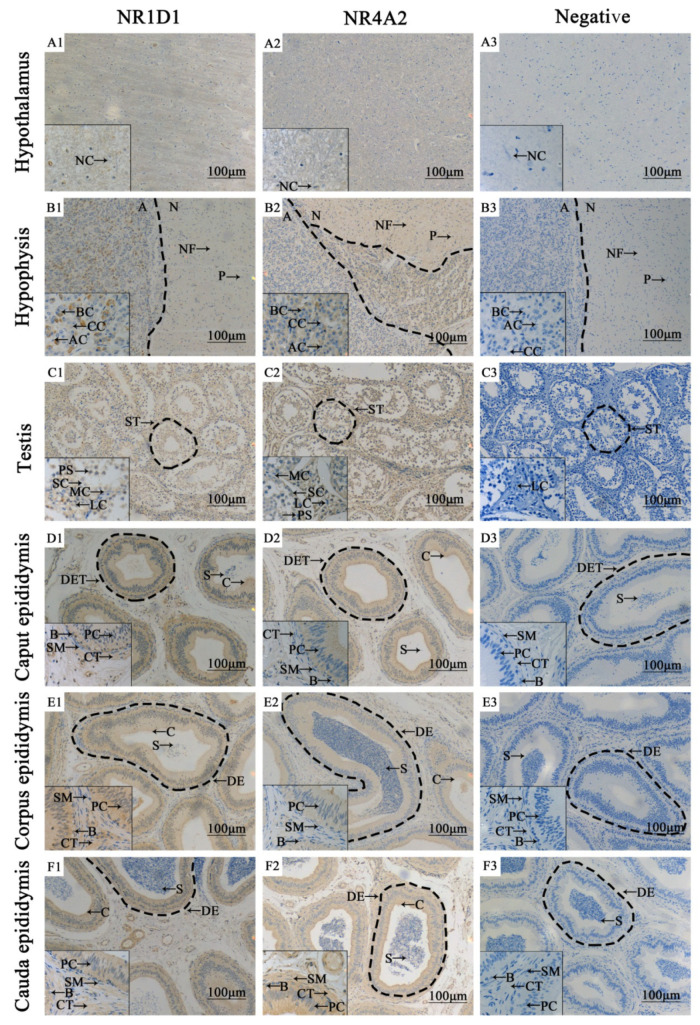
Localization of NR1D1 and NR4A2 proteins in adult male yak (4 years old) HPG tissues. (**A1**–**F1**), Localization of NR1D1 protein in adult male yak HPG tissues. (**A2**–**F2**), Localization of NR4A2 protein in adult male yak HPG tissues. (**A3**–**F3**), The negative control of adult male yak HPG tissues. NC, neurogliocyte (100×). The box in the lower left corner is a partial enlarged view (400×). A, adenohypophysis; N, neurohypophysis; P, pituicyte; AC, acidophilic cells; BC, basophilic cells; CC, chromophobe cells; NF, nerve fibers; ST, seminiferous tubule; MC, myoid cells; LC, interstitial cells; SC, spermatogonium; PS, primary spermatocyte; S, spermatozoon; DET, ductuli efferentes testis; B, basal cells; PC, principal cells; SM, smooth muscle cells; CT, connective tissue; DE, ductus epididymidis.

The subcellular location and expression analyses of NR1D1 and NR4A2 proteins in adult yak HPG tissues were performed using IHC. NR1D1 and NR4A2 proteins were found in all HPG tissues with differential expression patterns and staining degrees (Figure 2). In the hypothalamus, weak immuno-positive results were observed for NR1D1 and NR4A2 in the nerve fiber bundle, while the neurogliocytes remained unstained (Figure 2A1,A2). NR1D1 and NR4A2 proteins were found in the pituitary, mainly distributed in the cytoplasm of adenohypophysial basophilic cells and only present in nerve fibers of neurohypophysis (Figure 2B1,B2). In the neurohypophysis, the positive result for NR4A2 protein was more obvious than for NR1D1 protein. NR1D1 and NR4A2 proteins were also expressed in the cytoplasm of yak testicular tissues, particularly in Leydig cells and various spermatogenic cells (Figure 2C1,C2). The epididymal duct epithelium indicated expression of NR1D1 and NR4A2. In particular, the positive results in principal cells and cilia were more obvious than in other structures. A positive reaction for NR1D1 protein in the corpus epididymis was higher than in the caput epididymis or cauda epididymis (Figure 2D1,E1,F1). However, the NR4A2 protein showed strong immunoreactivity in the caput epididymis and only weakly in the corpus epididymis and cauda epididymis (Figure 2D2,E2,F2). The sperm in the lumen of the epididymis showed weak positive staining. The immuno-positive results for NR1D1 and NR4A2 proteins in the negative control group were not present in all yak HPG tissues (Figure 2A3,B3,C3,D3,E3,F3).

### 3.3. Localization of NR1D1 and NR4A2 in Adult Yak Testis and Epididymis Tissues

**Figure 3:**
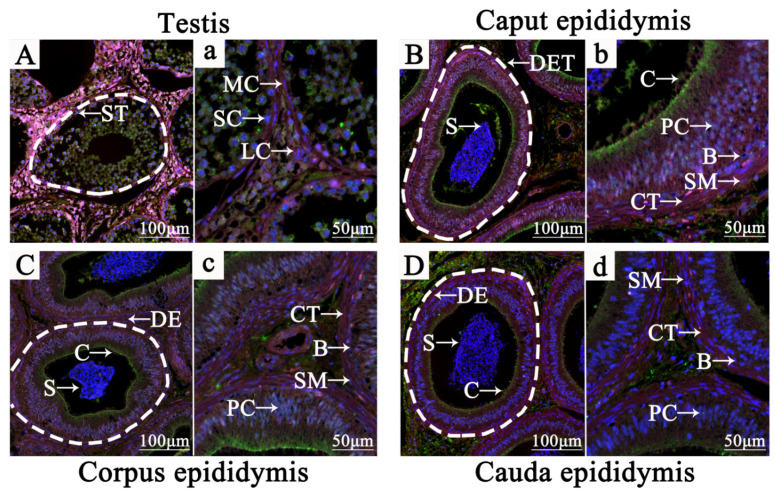
Localization of NR1D1 and NR4A2 in adult yak (4 years old) testis and epididymis tissues using IF staining. (**A**–**D**), localization of NR1D1 and NR4A2 in adult yak testis, caput epididymis, corpus epididymis and cauda epididymis, respectively (100×). (**a**–**d**), The amplification results of local structures in yak testis, caput epididymis, corpus epididymis and cauda epididymis, respectively (400×). ST, seminiferous tubule; MC, myoid cells; LC, interstitial cells; SC, spermatogonium; S, spermatozoon; DET, ductuli efferentes testis; B, basal cells; PC, principal cells; SM, smooth muscle cells; CT, connective tissue; DE, ductus epididymidis.

The IF signals of NR1D1, NR4A2 and 3β-HSD proteins were present in different cell types in adult yak testis and epididymis tissues, particularly in Leydig cells (Figure 3 and Supplementary Materials Figure S1). 3β-HSD, as a specific molecular marker of Leydig cells, was present in the cytoplasm of Leydig cells and where the NR1D1 and NR4A2 proteins were co-expressed (Figure 3A,a). In the caput epididymis, the IF signal of NR1D1, NR4A2 and 3β-HSD proteins were found mainly in the cytoplasm of the principal cells of the ductuli efferentes testis (DET) and smooth muscle cells. Interestingly, these proteins were co-expressed in the stereocilium of the DET with obvious differences (Figure 3B,b). NR1D1, NR4A2 and 3β-HSD proteins were expressed mainly in the principal cells cytoplasm and stereocilium of the ductus epididymidis (DE). NR1D1 was more strongly positive than NR4A2 protein in DE (Figure 3C,c). In the cauda epididymis, NR4A2 and 3β-HSD proteins were displayed prominently in the principal cells cytoplasm and stereocilium of DE. However, NR1D1 protein was found mainly in the connective tissue between the DE (Figure 3D,d). These results revealed that the functions of NR1D1 and NR4A2 proteins have a closed relationship with the reproductive hormones.

### 3.4. Expression Levels of NR1D1 and NR4A2 mRNA and Protein in HPG Tissues

**Figure 4:**
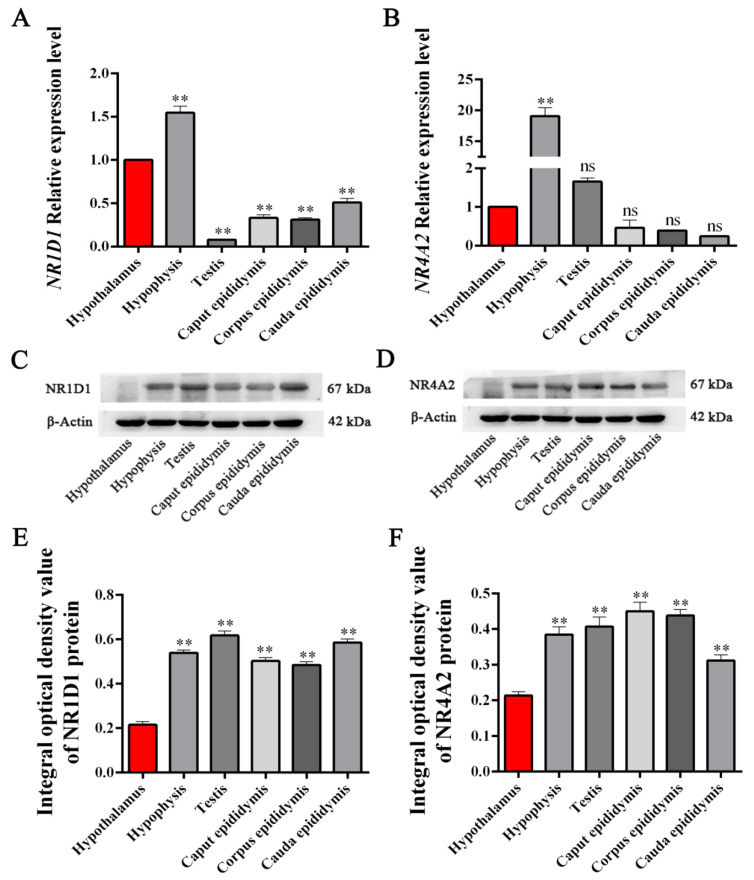
The expression levels of *NR1D1* and *NR4A2* mRNA and proteins in adult yak (4 years old) HPG tissues. (**A**,**B**), The expression of *NR1D1* and *NR4A2* mRNA, respectively, in yak HPG tissues, measured using qPCR. (**C**,**D**), The expression of NR1D1 and NR4A2 protein, respectively, in yak HPG tissues, measured using Western blot. (**E**,**F**), The IOD values of NR1D1 and NR4A2 proteins in yak HPG tissues, respectively. The expression levels of *NR1D1* and *NR4A2* mRNA and protein in the hypothalamus were used as the controls. The expression of β-actin was used as an endogenous control. **, represent extremely significant difference vs. control (*p* < 0.01).

The *NR1D1* and *NR4A2* mRNA and protein expression levels in yak HPG tissues were determined using qPCR and Western blot. The results showed that *NR1D1* and *NR4A2* mRNA and protein were significantly differentially expressed (*p* < 0.05, Figure 4A) in all yak reproductive axis tissues (Figure 4). Compared to the expression level of *NR1D1* mRNA in hypothalamus tissue, the highest expression levels of *NR1D1* mRNA were in the hypophysis tissue, followed by the cauda epididymis, corpus epididymis, caput epididymis and testis tissues. Compared to the expression level of *NR4A2* mRNA in the hypothalamus tissue, the highest expression levels of *NR4A2* mRNA were also in the hypophysis tissue, followed by testis tissue with a tendency of up-regulation. However, the expression of *NR4A2* mRNA in epididymis (caput, corpus and cauda) tissues were down-regulated compared to those in the hypophysis and hypothalamus and without significant difference (Figure 4B). The relative expression levels of NR1D1 and NR4A2 proteins were detected in yak reproductive axis tissues with some differences (Figure 4C,D). The average integral optical density (IOD) value of the bands was applied to evaluate the differential expression levels. The results showed that NR1D1 and NR4A2 proteins in the hypophysis, testis and epididymis (caput, corpus and cauda) tissues were significantly differentially up-regulated compared to hypothalamus tissue (*p* < 0.05). The highest expression levels of NR1D1 protein were in testis tissue, while the highest expression levels of NR4A2 protein were found in caput epididymis tissue (Figure 4E,F).

### 3.5. Expression Patterns of NR1D1 and NR4A2 mRNA and Protein in Yak Testicular Tissues with Different Ages

**Figure 5:**
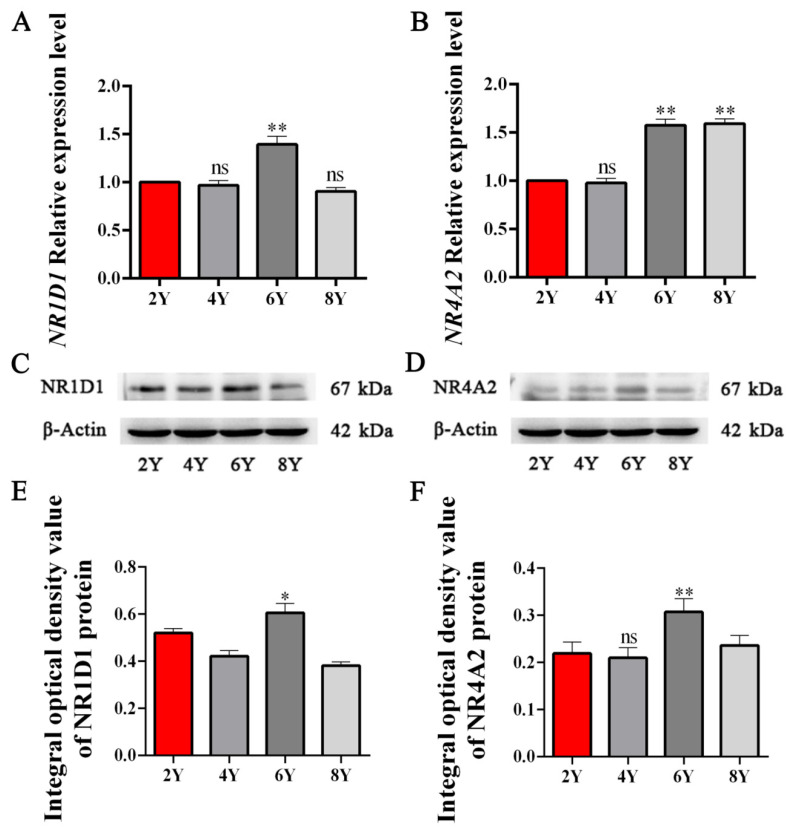
Expression patterns of *NR1D1* and *NR4A2* mRNA and protein in yak testicular tissues in animals of different ages. (**A**,**B**), The expression of *NR1D1* and *NR4A2* mRNA, respectively, in yak testicular tissues from animals of different ages, detected using qPCR. (**C**,**D**), The expression of NR1D1 and NR4A2 protein, respectively, detected using Western blot in yak reproductive axis tissues. (**E**,**F**), The IOD value of NR1D1 and NR4A2 protein, respectively, in testicular tissues from yaks of different ages. The expression levels of *NR1D1* and *NR4A2* mRNA and protein in testicular tissues of 2-year-old yaks were used as controls. The expression of β-actin was used as an endogenous control. *, represent significant difference vs. control (*p* < 0.05), **, represent extremely significant difference vs. control (*p* < 0.01). Y, Year; ns, represent NO significant difference.

The mRNA and protein expression levels of *NR1D1* and *NR4A2* were also examined in yak testicular tissues from yaks of different ages (Figure 5). The results showed that *NR1D1* and *NR4A2* mRNA and protein were differentially expressed in yak testicular tissues at different ages. Compared to the expression levels of *NR1D1* and *NR4A2* mRNA in testicular tissues from 2-year-old yaks, the highest expression levels of *NR1D1* and *NR4A2* mRNA were present in testicular tissues of yaks at 6 years of age and the difference was significant (*p* < 0.01, Figure 5A,B). The expression level of *NR4A2* mRNA was also significantly different in testicular tissues of yaks at 8 years of age (*p* < 0.01). The results showed that NR1D1 and NR4A2 proteins were differentially expressed in yak testicular tissues at different ages (Figure 5C,D). Using the testicular tissues of yaks at 2 years of age as a control, the highest expression levels of NR1D1 and NR4A2 proteins were also measured in testicular tissues of 6-year-old yaks (*p* < 0.01, Figure 5E,F).

### 3.6. GO Functional and Pathway Analyses of Yak NR1D1 and NR4A2 in Reproductive Hormone Metabolism

**Figure 6:**
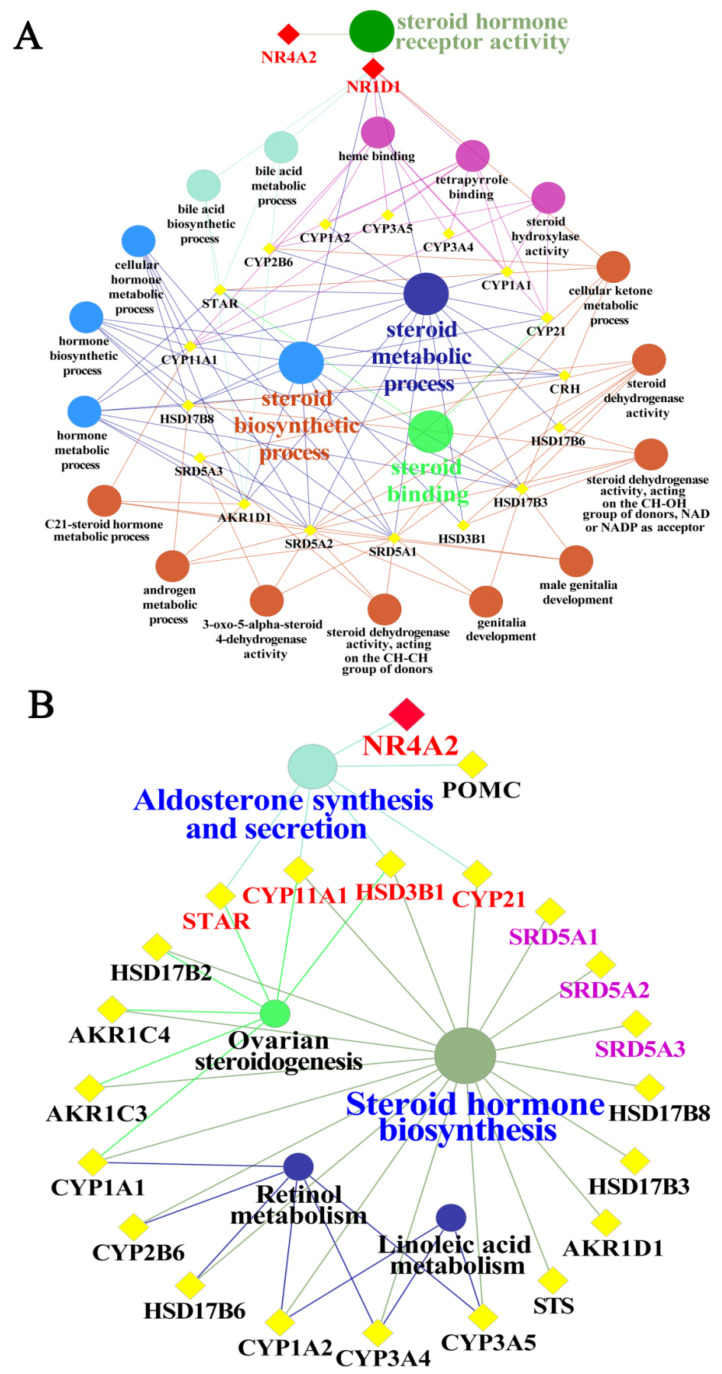
GO and pathway prediction of yak NR1D1 and NR4A2 in reproductive hormone metabolism. (**A**), Network of GO terms and associated proteins in reproductive hormone metabolism. (**B**), Network of pathways and associated proteins in reproductive hormone metabolism.

The results of GO functional enrichment and KEGG pathway analyses demonstrated that NR1D1 and NR4A2 directly or indirectly participated in various biological processes associated with reproductive hormone metabolism, and particularly in steroid hormone metabolism (Figure 6). According to the GO functional analysis, NR4A2 and NR1D1 may directly regulate steroid hormone receptor activity. The NR1D1 participates widely in reproductive hormone processes, such as the steroid biosynthetic process, steroid metabolic process and cellular ketone metabolic process (Figure 6A). In addition, NR1D1 regulates indirectly some important proteins or rate-limiting enzymes that regulate the synthesis and metabolism of androgens, including testosterone and dihydrotestosterone, steroidogenic acute regulatory protein (StAR), steroid 5 alpha-reductase 1 (SRD5A1), steroid 5 alpha-reductase 2 (SRD5A2) and hydroxy-delta-5-steroid dehydrogenase-3 beta (HSD3B1). Additionally, the results of pathway analysis described that NR4A2 participated directly in regulating aldosterone synthesis and secretion. NR4A2 participated indirectly in ovarian steroidogenesis, steroid hormone biosynthesis via regulating the HSD3B1 and cytochrome P450, family 11, subfamily A, and polypeptide 1 (CYP11A1). NR4A2 participated indirectly in the metabolism of linoleic acid and retinol that affects the synthesis and metabolism of reproductive hormones in animals (Figure 6B). Taken together, we confirmed that NR1D1 and NR4A2 can directly or indirectly regulate the synthesis and metabolism of reproductive hormones.

## 4. Discussion

The reproductive function and behaviors of male animals are regulated by HPG tissues that are responsible for hormone synthesis and secretion. Steroid hormones, a class of reproductive hormones that includes some important reproductive hormones such as androgens and estrogens, are thought to be strictly regulated by HPG tissues via gene expression [25]. Understanding the histomorphology and physiological functions of HPG tissues is vital for elucidating the molecular mechanisms of animal reproduction. Previous research has elaborated greatly on the molecular mechanism of steroid hormones and their related genes or proteins [26,27]. However, the topic remains incompletely explored. NRs, as a kind of transcription factor, are recruited to some ligands to convey and translate signals for the regulation of hormone-related gene expression. Thus, it is essential to illuminate the distribution, expression and function of the NRs in reproductive axis tissues. Several nuclear receptors have been shown to play essential roles in animal reproduction [11,17], but NR1D1 and NR4A2 have been lacking in identified target genes and related molecular mechanisms, particularly in yaks. NR1D1 and NR4A2, members of the NR superfamily, are important receptors of hormones. It remains unknown whether or not NR1D1 and NR4A2 participate in the regulation of yak reproductive hormones.

In the present study, we observed the histomorphology of HPG tissues from male Tianzhu white yaks using H&E staining. The results showed that the main HPG tissue structures appeared structures of were intact (Figure 1). Subsequently, we found that NR1D1 and NR4A2 proteins were present in all yak HPG tissues, particularly in the adenohypophysis, Leydig cells, principal cells and cilia of epididymis (Figure 2). Previous studies have demonstrated that NR1D1 and NR4A2 proteins were expressed in these tissues in other species [28,29,30]. The androgens synthesized by Leydig cells, luteinizing hormone secreted by adenohypophysis, and steroid hormones secreted and absorbed by epididymis cilia are important for animal reproduction, particularly in the maturation of germ cells [31,32]. It has been suggested that NR1D1 and NR4A2 may participate in yak endocrine regulation because the functions of these tissues or cells are responsible mainly for animal reproduction and especially for hormone synthesis and metabolism. IF results revealed that the NR1D1 and NR4A2 proteins were co-located in Leydig cells and cilia of epididymis (Figure 3). As mentioned previously, the main function of Leydig cells is the secretion of androgens, including testosterone and dihydrotestosterone, that are strictly regulated by HPG tissues [33,34] and are related to genes or enzymes such as HSD3B, CYP17A1 and StAR. It has been shown that NR1D1 and NR4A2 can activate or suppress the expression of genes involved in testosterone biosynthesis in Leydig cells [14,32,35].

Moreover, the high expression of NR1D1 and NR4A2 proteins in principal cells and cilia of epididymis were dependent on the function of the epididymis. Because the primary modulators of the epididymis are androgens, 5α-reduced metabolite of testosterone and 5α-dihydrotestosterone are crucial for a series of changes in sperm functions (i.e., sperm motility and maturation) [36]. 

The results of qPCR and Western blot showed that the highest expression levels of *NR1D1* and *NR4A2* mRNA were in the hypophysis, whereas the NR1D1 and NR4A2 proteins were differentially expressed in all yak reproductive axis tissues (Figure 4). The hypothalamus-hypophysis, as the central control system of animal biological processes, receives and responds to biological signals prior to the gonad axis (testis and epididymis for males), especially in endocrine processes [37,38]. It was revealed that NR1D1 and NR4A2, as transcription factors, could deliver the biological signals from the hypophysis to the gonad axis. The results of qPCR and Western blot in yak testicular tissues from animals of different ages confirmed that the expression levels of NR1D1 and NR4A2 were up- or down-regulated with the maturation or decline of sexual function (Figure 5). It was suggested that the expression of NR1D1 and NR4A2 in yak testicular tissues tended to be weak during sexual immaturity and strongest in the most vigorous mating ability (3–7 years old).

The results of GO and pathway enrichment analyses showed that NR1D1 and NR4A2 interacted directly or indirectly with proteins related to steroid hormone biosynthesis, especially StAR, HSD3B and SRD5A (Figure 6), which are rate-limiting enzymes of steroidogenesis. For instance, StAR protein translocates cholesterol from the outer to the inner mitochondrial membrane and is used further for steroid hormone biosynthesis in steroidogenic cells [39]. Testosterone is converted into 5α-dihydrotestosterone by SRD5A in steroidogenic tissues and peripheral tissues [40]. The expression patterns, locations, potential functions and regulatory networks of NR1D1 and NR4A2 were elaborated in the present study, but the specific regulation of NR1D1 and NR4A2 in hormone metabolism requires further study. These results will help to improve the understanding of the reproductive physiological characteristics and to further improve the reproductive performance of male yaks.

## 5. Conclusions

In the present study, we described the morphological structures of HPG tissues in male Tianzhu white yaks. The NR1D1 and NR4A2 proteins were present in all yak HPG tissues, particularly in the adenohypophysis, Leydig cells, principal cells and cilia of the epididymis. The NR1D1 and NR4A2 proteins were mainly co-located in Leydig cells and the cilia in the epididymis. The *NR1D1* and *NR4A2* mRNAs and proteins were differentially expressed in all yak reproductive axis tissues and were particularly highly expressed in the hypophysis. The expression levels of *NR1D1* and *NR4A2* mRNAs and proteins in the different testicular tissues were up- or down-regulated with the maturation or decline of sexual function in yaks. NR1D1 and NR4A2 interacted, either directly or indirectly, with proteins related to steroid hormone biosynthesis. These results suggested that NR1D1 and NR4A2 are involved in the regulation of steroid hormones and androgen metabolism in male yaks.

## Data Availability

The data that support the findings of this study are available from the corresponding author upon reasonable request.

## Supplementary Materials

The following are available online at https://www.mdpi.com/article/10.3390/ani11113117/s1, Table S1: qPCR primer sequences, Table S2: The candidate genes or proteins related to reproductive hormone metabolism, Figure S1: Localization of NR1D1, NR4A2 and 3β-HSD in adult yak testis and epididymis tissues using IF staining.
