# Supplementary material for: The Distribution, Expression Patterns and Functional Analysis of NR1D1 and NR4A2 in the Reproductive Axis Tissues of the Male Tianzhu White Yak
Source: Animals (Basel). 2021 Oct 31;11(11):3117. doi: 10.3390/ani11113117 (PMC8614427; doi:10.3390/ani11113117)
Supplement: Supplementary file 1 [file animals-11-03117-s001.zip › animals-1417818-supplementary.pdf]

Table S1: qPCR primer sequences.

Gene	Primer sequence (5'-3')	GenBank accession	Annealing temperature (℃)	Amplicon size (bp)
NR1D1	F: ATGACGACCCTGGACTCTAA<br>R: GTGATGGTGGGAAGTAGGTG	XM_019981024.1	59℃	166 bp
NR4A2	F: GTTCAGGCGCAGTATGGG<br>R: GTGGCAGTGATTTCAGTGTT	XM_027560333.1	59℃	152 bp
β-Actin	F: AACCGTGAGAAGATGACCCA<br>R: GGCGTACCCCTCGTAGATG	XM_027528015.1	59℃	168 bp

Table S2: Signal pathway related genes.

NO.	Gene ID	Gene symbol	Description
1	102264589	STS	steroid sulfatase
2	531192	SULT2B1	sulfotransferase family 2B member 1
3	338048	CYP11A1	cytochrome P450, family 11, subfamily A, polypeptide 1
4	281739	CYP17A1	cytochrome P450, family 17, subfamily A, polypeptide 1
5	281824	HSD3B1	hydroxy-delta-5-steroid dehydrogenase, 3 beta- and steroid delta-isomerase 1
6	617917	AKR1C3	aldo-keto reductase family 1, member C3 (3-alpha hydroxysteroid dehydrogenase, type II)
7	102284785	HSD17B3	hydroxysteroid 17-beta dehydrogenase 3
8	102278674	HSD17B2	hydroxysteroid 17-beta dehydrogenase 2
9	102283510	HSD17B6 / LOC102283510	17-beta-hydroxysteroid dehydrogenase type 6
10	102286352	HSD17B8	hydroxysteroid 17-beta dehydrogenase 8
11	338042	UGT / SLC35A2	solute carrier family 35 member A2
12	282870	CYP1A1	Cytochrome P450, subfamily I (aromatic compound-inducible), polypeptide 1
13	503552	CYP1A2	cytochrome P450, family 1, subfamily A, polypeptide 2
14	504769	CYP2B / CYP2B6	cytochrome P450 subfamily 2B
15	100328933	CYP2C	cytochrome P450-like
16	282211	CYP2D14	cytochrome P450, family 2, subfamily D, polypeptide 6
17	101155406	Cyp3a	cytochrome P450, family 3, subfamily A
18	282214	CYP3A5	cytochrome P450, subfamily IIIA (niphedipine oxidase), polypeptide 4
19	1551	CYP3A7	cytochrome P450 family 3 subfamily A member 7
20	507988	CYP3A4	cytochrome P450, subfamily IIIA, polypeptide 4
21	282213	CYP2E1	cytochrome P450, family 2, subfamily E, polypeptide 1

22	102273138	SRD5A1	steroid 5 alpha-reductase 1
23	102271230	SRD5A2	steroid 5 alpha-reductase 2
24	102284607	SRD5A3	steroid 5 alpha-reductase 3
25	282138	AKR1C4	aldo-keto reductase family 1, member C4 (chlordecone reductase; 3-alpha hydroxysteroid dehydrogenase, type I; dihydrodiol dehydrogenase 4)
26	102264964	AKR1D1	aldo-keto reductase family 1 member D1
27	102281340	INSL3	insulin like 3
28	102277345	STAR	steroidogenic acute regulatory protein
29	102273696	CRH	corticotropin releasing hormone
30	281741	CYP21	cytochrome P450, subfamily XXI (steroid 21-hydroxylase)
31	102270158	AR	androgen receptor
32	102283025	NR1D1	nuclear receptor subfamily 1 group D member 1
33	102277637	NR4A2	nuclear receptor subfamily 4 group A member 2

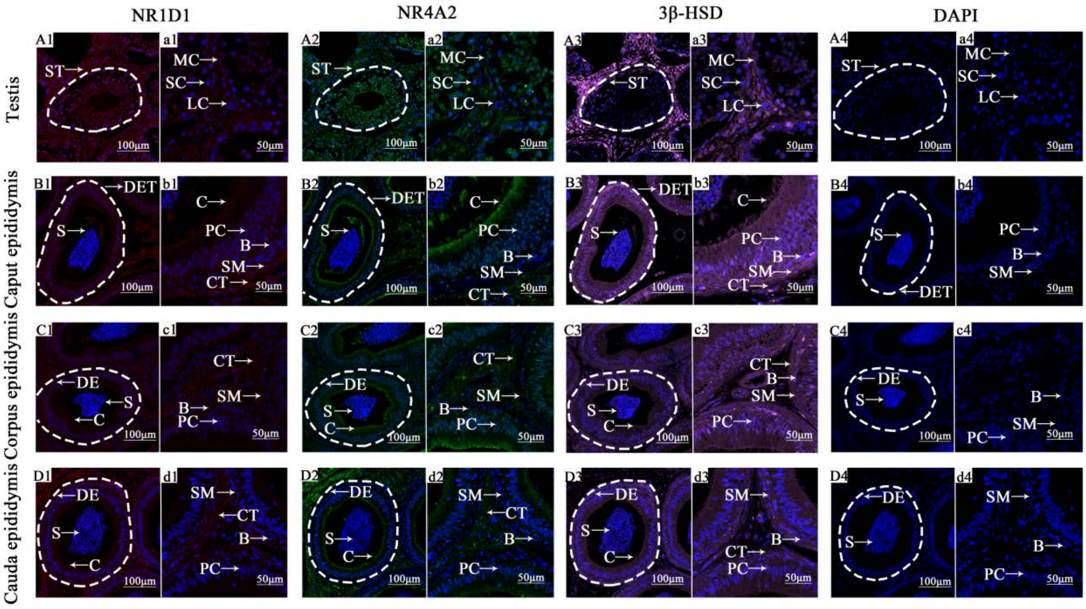

Figure S1. Localization of NR1D1, NR4A2 and 3β-HSD in adult yak testis and epididymis tissues using IF staining. Figure 3. Localization of NR1D1, NR4A2 and 3β-HSD in adult yak (4 years old) testis and epididymis tissues using IF staining. A1 to D1, Localization of NR1D1 protein in adult yak testis, caput epididymis, corpus epididymis and cauda epididymis. A2 to D2, Localization of NR4A2 protein in adult yak testis, caput epididymis, corpus epididymis and cauda epididymis. A3 to D3, Localization of 3β-HSD protein in adult yak testis, caput epididymis, corpus epididymis and cauda epididymis. A4 to D4, The negative control of adult yak testis, caput epididymis, corpus epididymis and cauda epididymis. NC, neurogliocyte (100×). The picture on the left with lowercase letters magnifies the field of view (400×). ST, seminiferous tubule. MC, myoid cells. LC, interstitial cells. SC, spermatogonium. S, spermatozoon. DET, ductuli efferentes testis. B, basalbasic cells. PC, principal cellsmaster cell. SM, smooth muscle cells. CT, connective tissue. DE, ductus epididymidis.
